# The relationship between oxidative stress, reproduction, and survival in a bdelloid rotifer

**DOI:** 10.1186/s12898-019-0223-2

**Published:** 2019-02-01

**Authors:** Leigh C. Latta, K. Nathaniel Tucker, Robert A. Haney

**Affiliations:** 1 Division of Natural Sciences and Mathematics, Lewis-Clark State College, 500 8th Avenue, Lewiston, ID 83501 USA; 2 Department of Molecular and Cellular Biology, Harvard University, Cambridge, MA 02138 USA; 3 Department of Biological Sciences, University of Massachusetts Lowell, Lowell, MA 01854 USA

**Keywords:** *Adineta vaga*, Ionizing radiation, Life-history, Oxidative stress

## Abstract

**Background:**

A proposed mediator of trade-offs between survival and reproduction is oxidative stress resistance. Investments in reproduction are associated with increased oxidative stress that reduces lifespan. We used the bdelloid rotifer *Adineta vaga* to examine baseline patterns of survival, reproduction, and measures of oxidative stress, as well as how these patterns change in the face of treatments known to induce oxidative stress.

**Results:**

We discovered that under standard laboratory conditions late-life mortality may be explained by increased levels of oxidative stress induced by reproduction. However, following exposure to the oxidizing agent ionizing radiation, survival was unaffected while reproduction was reduced.

**Conclusions:**

We suggest that under normal environmental conditions, reduced survival is mediated by endogenously generated oxidative stress induced by reproduction, and thus represents a cost of reproduction. Alternatively, the reduced reproduction evident under exogenously applied oxidative stress represents a cost of somatic maintenance. Biochemical analyses designed to assess levels of oxidative stress, oxidative stress resistance, and oxidative damage under normal and oxidizing conditions suggest that varying investments in enzymatic and non-enzymatic based oxidative stress resistance determine whether a cost of reproduction or a cost of somatic maintenance is observed.

**Electronic supplementary material:**

The online version of this article (10.1186/s12898-019-0223-2) contains supplementary material, which is available to authorized users.

## Background

A focus of evolutionary ecology research is identifying the physiological mechanisms that underlie life-history trade-offs [1]. Recently, it has been hypothesized that oxidative stress, an imbalance between the production of reactive oxygen species (free radicals) and antioxidant defense, provides a mechanism for trade-offs between reproduction and somatic maintenance [2–5]. The premise of this hypothesis is that organisms faced with increased levels of oxidative stress can invest resources into somatic maintenance (the protection and repair of biomolecules susceptible to, or damaged by, oxidative attack) that should enhance survival and longevity, but at the expense of investments in other energetically demanding functions such as reproduction. Alternatively, organisms may ignore the threat of oxidative damage, thus risking decreased survival and longevity, and instead invest resources into functions like reproduction.

Unfortunately, much of the research on the proposed relationship between oxidative stress and life-history trade-offs has produced inconclusive results [6]. Some inconsistencies within and among studies may be the result of an incomplete picture of the oxidative stress system, which includes not only characterizations of the enzymatic and non-enzymatic antioxidant defenses, but also levels of oxidative stress (e.g., superoxide anions, hydrogen peroxide, and hydroxyl radicals) and oxidative damage (e.g., protein carbonylation and lipid peroxidation). Additional criticisms of the experiments intended to test the hypothesis include designs that do not artificially manipulate levels of reproduction, provide resources ad libitum, or measure only segments of organismal lifespan [6, 7].

An additional consideration of experiments designed to test the oxidative stress hypothesis involves the methods and interpretation of analysis. Statistically, a life-history trade-off in the context of this hypothesis is indicated by a significant negative correlation between measures of survival and reproduction. Appropriately, some studies have revealed a trade-off between survival and reproduction following interventions designed to modify resource availability or allocation [1, 8]. However, it has recently been proposed that oxidative stress can still mediate the relationship between traits even in the absence of trade-offs [9]. In particular, positive correlations between survival and reproduction that are significantly reduced in strength following environmental manipulations that alter levels of oxidative stress provide evidence that oxidative stress mediates the relationship between survival and reproduction, even in the absence of a trade-off sensu stricto.

In these experiments we use the bdelloid rotifer, *Adineta vaga*, as a model to examine the physiological basis of the relationship between survival and reproduction. Previous results show that *A. vaga* survival and reproduction respond differently to ionizing radiation (IR) [10, 11], the effects of which primarily stem from free radical production due to the ionization of intracellular water molecules. Of particular interest is the observation that bdelloid fecundity is more sensitive to IR-induced free radical formation than survival, with the IR dose required to reduce fecundity about ten times less than that required to reduce survival [11]. Therefore, exposure to IR provides a means to experimentally manipulate fecundity. The heightened sensitivity of reproduction to IR also suggests bdelloids may prioritize oxidative stress resistance for the purpose of somatic maintenance over reproduction when faced with environmental stress. The implication is that rather than costs of reproduction (such as increased oxidative damage that reduces lifespan) driving the relationship between survival and reproduction, it is costs of somatic maintenance (reduced reproduction in order to maintain oxidative stress resistance) that are critical.

Other features of bdelloid biology position them to address the oxidative stress hypothesis. First, bdelloids are eutelic, with all somatic and germline cell divisions finished prior to hatching from the egg [12–14]. Thus, increases in body size that occur as a juvenile or adult involve only changes in cell size. This aspect of development means that any possible trade-offs involving growth rate that may confound a trade-off between reproduction and survival will only be due to resources allocated to changes in cell size, and not resources allocated to cell division. Also, bdelloids reproduce asexually which allows replication of genotypes and evaluation of genotype-specific fitness across environments.

Here, we examine the relationship between patterns of age-specific survival and fecundity in *A. vaga* under standard laboratory conditions and following exposure to environmental stress that modifies reproduction (IR). In conjunction with life-table experiments, we also used biochemical assays that measure levels of oxidative stressors (H_2_O_2_), levels of enzymatic-based (catalase and peroxidase activity) and non-enzymatic-based (small-molecule antioxidants) oxidative stress resistance, and levels of oxidative damage (protein carbonylation). Combined, these experiments overcome some of the experimental design limitations that may have confounded previous investigations because we employ IR as a means to experimentally manipulate reproduction, we measure all components of the oxidative stress system (oxidative stress, oxidative stress resistance, and oxidative damage), and we measure these in conjunction with estimates of survival and reproduction over the entire lifespan of the individuals. An additional criticism of experiments designed to test the oxidative stress hypothesis revolve around providing resources ad libitum [6]. It is still unclear whether limited extrinsic resources are more important than intrinsic physiological factors in determining levels of reproduction [7], so we use the traditional laboratory approach of feeding ad libitum with the caveat that our results might be altered under different feeding regimes.

With these experiments we first establish baseline expectations for changes in survival, reproduction, and oxidative stress biology as a function of age under standard laboratory conditions. We then examine how the relationship between survival and reproduction changes following experimental manipulation of reproduction through IR exposure. If oxidative stress underlies the relationship between survival and fecundity then we predict that the strength and/or sign of the correlation between these traits measured under standard laboratory conditions will significantly change in response to the experimental manipulation of reproduction. Finally, we characterize the biochemical basis of protection against IR induced free radicals in order to understand the physiological basis of changes in survival and fecundity.

## Results

### Baseline

#### Survival and reproduction

**Fig. 1:**
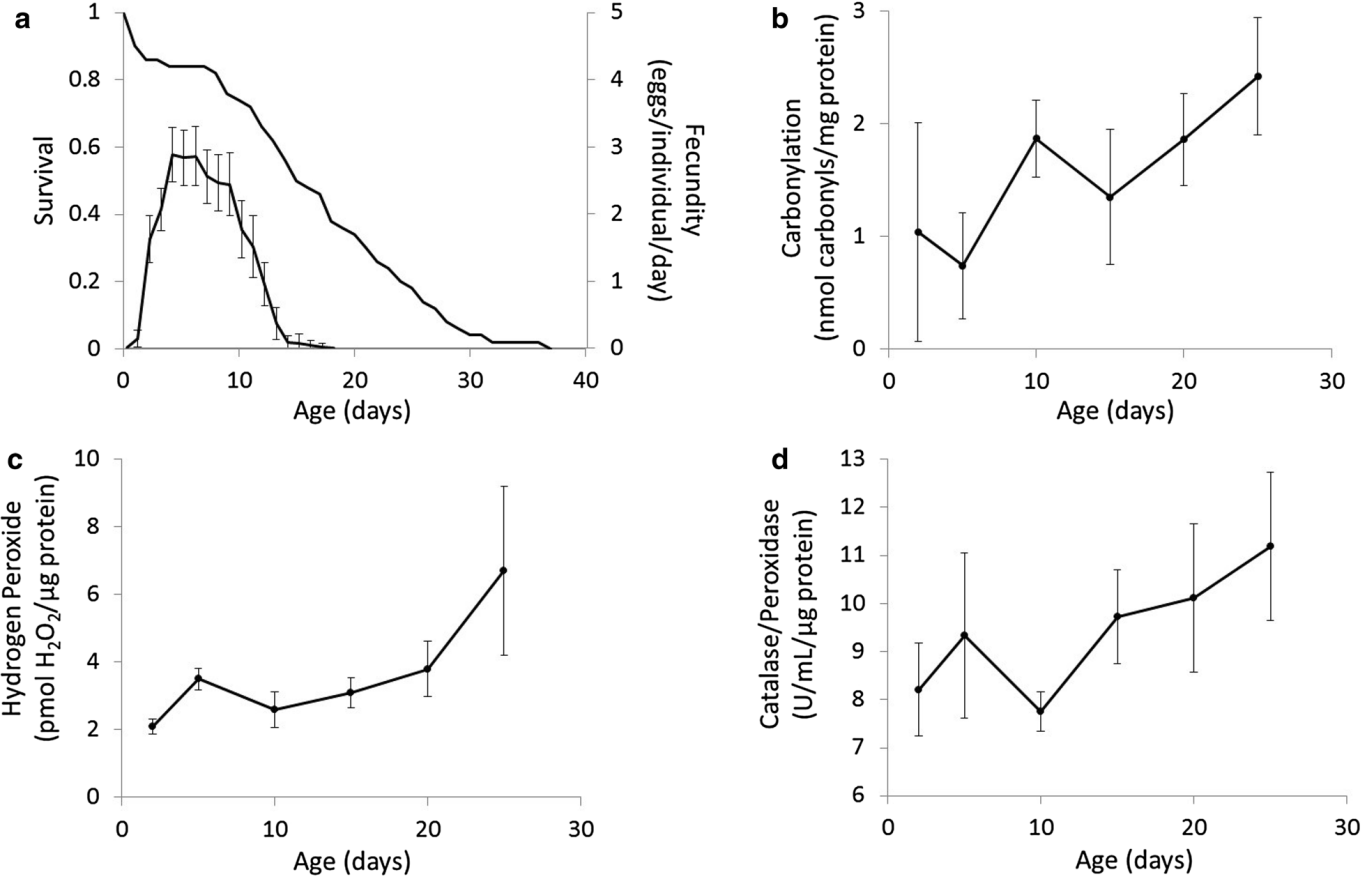
Age-specific changes in life-history and biochemistry. **a** Age-specific survival and fecundity estimated from a life-table experiment (n = 72 individuals). Age-specific levels of **b** oxidative damage measured as protein carbonylation, **c** the oxidizing agent hydrogen peroxide, and **d** enzymatic-based oxidative stress resistance measured as catalase/peroxidase activity. Error bars are ± 2 s.e.m

Our measurements of age-specific patterns of survival and reproduction under benign laboratory conditions in *A. vaga* show that peak reproduction occurs between the ages of 5 and 10 days (Fig. 1a). Roughly concurrent with the early stages of peak reproduction is a survival plateau between the ages of 2 and 7 days. By age 8 days, the age-specific mortality rate increases and survival declines monotonically until late in life beyond age 30 days.

These data indicate that the mean lifespan of *A. vaga* in our life-table experiment was 16.2 days, with a median of 15.5 days, and a maximum of 37 days. Fitting a Gompertz model to the survival data of *A. vaga* yields an estimate of the mortality rate doubling time of 8.36 days. The net reproductive rate is 21.26 eggs per individual lifetime. Combined, the survival and fecundity data (Fig. 1a) indicate the intrinsic rate of increase is 0.45 days^−1^. Finally, we identified a significant positive correlation between reproduction and lifespan (r = 0.80; 95% CI 0.67–0.88) for individuals reared under standard laboratory conditions.

#### Oxidative stress biochemistry

Our age-specific estimates of the levels of oxidative damage, oxidative stress, and oxidative stress resistance indicate that all three components of the oxidative stress system tend to increase with age. Specifically, one-factor ANOVA on age-specific protein carbonylation revealed a significant effect of age (F = 4.44; df = 5; p = 0.003) with protein carbonylation generally increasing with age (Fig. 1b). Specifically, post hoc pairwise comparisons with p-value adjustments to account for multiple comparisons indicated that 25-day-old individuals had significantly higher levels of protein carbonylation than 2-day-old individuals (t ratio = 1.48; df = 36; p = 0.023) and 5-day-old individuals (t ratio = 4.05; df = 36; p = 0.003). One-factor ANOVA on age-specific H_2_O_2_ levels also showed a significant effect of age (F = 7.40; df = 5; p < 0.001) with levels generally increasing with increased age (Fig. 1c). Pairwise comparisons indicated that 25-day-olds had significantly higher levels of H_2_O_2_ than 2-day-olds (t ratio = 5.05; df = 28; p < 0.001), 5-day-olds (t ratio = 3.95; df = 28; p = 0.006), 10-day-olds (t ratio = 5.02; df = 28; p < 0.001), 15-day-olds (t ratio = 4.38; df = 28; p = 0.002), and 20-day-olds (t ratio = 3.46; df = 28; p = 0.020). Finally, ANOVA on age-specific catalase/peroxidase activity showed a main effect of age (F = 3.76; df = 5; p < 0.010), although the change in enzyme activity with age does not appear to be a general increase (Fig. 1d). Indeed, post hoc pairwise comparisons indicated that the only significant difference was higher levels of catalase/peroxidase activity in 25-day-olds relative to 10-day-olds (t ratio = 3.69; df = 28; p = 0.011).

### Ionizing radiation

#### Survival and reproduction

**Fig. 2:**
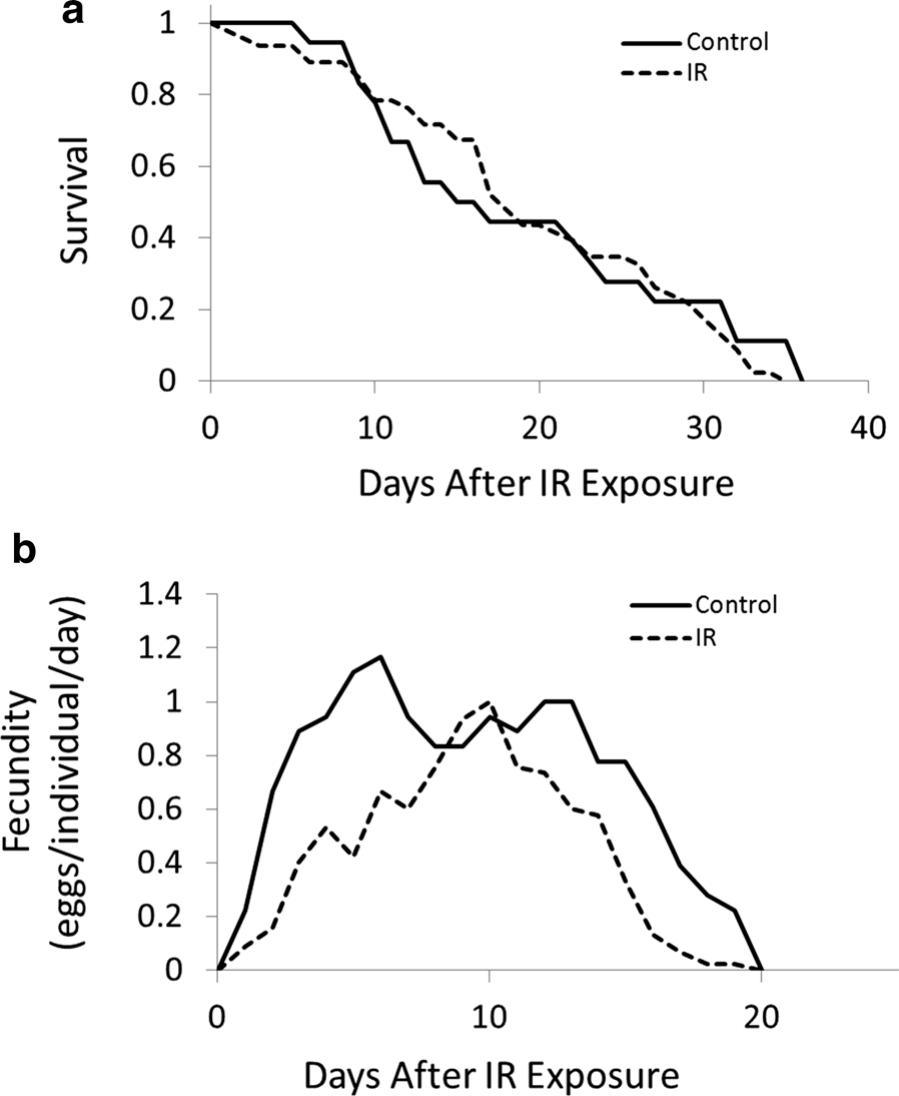
Age-specific patterns of **a** survival and **b** fecundity for non-irradiated (control; n = 18 individuals) and irradiated (IR; dose = 1200 Gy; n = 46 individuals) cohorts of *A. vaga* initiated from pre-reproductive individuals (1–4 days old)

Exposure to IR did not alter patterns of survival, but did change age-specific schedules of reproduction in *A. vaga*. Cox regression suggested no difference in the survival curves between irradiated and non-irradiated cohorts (Fig. 2a; Hazard Ratio = 1.15; regression coefficient = 0.14; s.e. = 0.29; p = 0.64). Our generalized linear model indicated a significant change in patterns of age-specific fecundity in response to ionizing radiation (regression coefficient = − 0.57; s.e. = 0.10; z-value = − 5.85; p < 0.001), with irradiated rotifers experiencing reduced fecundity for all age classes except ages 8–11 days, and overall (Fig. 2b; control = 14.5 eggs/individual, 2 s.e.m. = 3.9; irradiated = 8.8 eggs/individual, 2 s.e.m. = 1.83). The model also indicated significant effects of age (regression coefficient = 0.54; s.e. = 0.04; z-value = 13.56; p < 0.001) and age^2^ (regression coefficient = − 0.03; s.e. = 0.00; z-value = − 14.34; p < 0.001).

Exposure to IR also significantly altered the nature of the correlation between survival and reproduction. Specifically, the correlation between survival and reproduction in the irradiated cohort (r = 0.67; 95% CI 0.47–0.80) is significantly reduced (z = 1.86; p = 0.031) relative to the non-irradiated cohort (r = 0.88; 95% CI 0.70–0.95).

#### Oxidative stress biochemistry

**Fig. 3:**
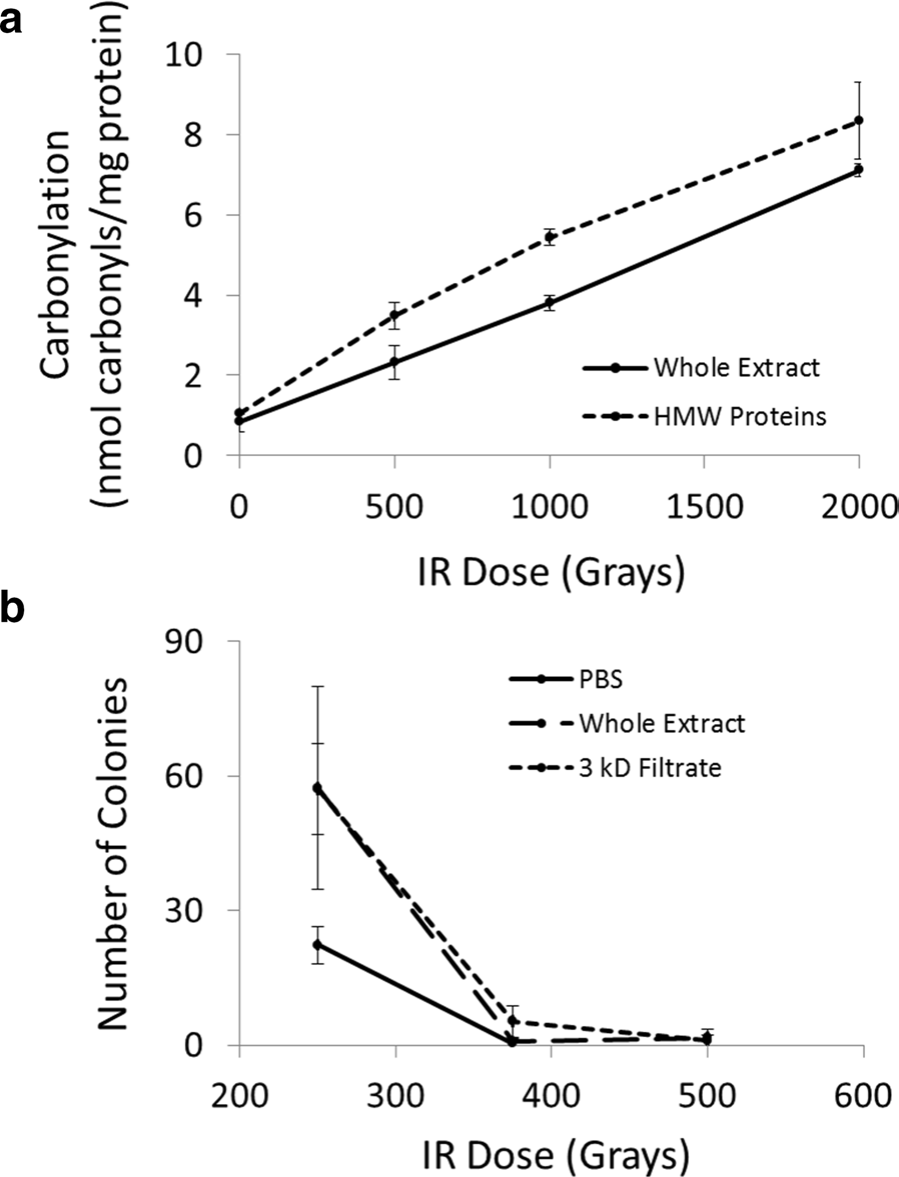
Small molecule antioxidant protection of native proteins and live cells. **a** Levels of protein carbonylation in extracts that contain proteins and small molecule antioxidants (Whole Extract) or only high molecular weight proteins (HMW Proteins) in response to IR. **b** Survival of *E. coli* cells irradiated in liquid culture supplemented with no additional antioxidants (PBS), proteins and small molecule antioxidants isolated from *A. vaga* (Whole Extract), or small molecule antioxidants isolated from *A. vaga* (3 kD Filtrate). Error bars are ± 2 s.e.m

Our assays designed to assess the effectiveness of non-enzymatic oxidative stress resistance in *A. vaga* suggested that the cocktail of small-molecule antioxidants is highly effective at protecting proteins from oxidative damage, and also at protecting live bacterial cells from the lethal effects of IR. The protein concentration of whole extracts and high molecular weight protein extracts exposed to radiation to measure protein carbonylation was 0.85 mg/mL. Two-factor ANOVA indicated a significant difference between the extracts (F = 52.52; df = 1, 16; p < 0.001) with high molecular weight extracts exhibiting higher levels of carbonylation than whole extracts (Fig. 3a). Carbonylation increased in response to IR dose (F = 388.86; df = 3, 16; p < 0.001) and there was a significant extract × dose interaction (F = 4.28; df = 3, 16; p = 0.021).

The protein concentration of whole extracts and filtrates derived from extracts for CFU assays of live *Escherichia coli* was 0.71 mg/mL. Generalized modeling indicated a significant effect of extract type (Deviance = 55.2; df = 2, 24; p < 0.001) with PBS only (which contained no small-molecule antioxidants) resulting in higher cell mortality following exposure to IR than whole extract or filtrate (Fig. 3b). There was a significant effect of IR dose (Deviance = 544.3; df = 2, 22; p < 0.001) with the number of surviving colonies decreasing as a function of increasing dose, and a significant extract × dose interaction (F = 16.32; df = 4, 18; p = 0.002).

## Discussion

Trade-offs between reproduction and somatic maintenance feature prominently in life-history evolution theory. One hypothesis that describes the physiological basis of these trade-offs invokes oxidative stress as the mediator [3]. However, experimental tests of this hypothesis have recently been criticized [7], and some inconsistencies among results in different systems may reflect issues with the experimental design [6]. Trade-offs between traits are indicated by negative phenotypic correlations, however, trade-offs between survival and reproduction are not strictly necessary to invoke oxidative stress as a mediator of relationships between traits. An alternative is to compare the strength of the correlation between traits measured under normal conditions and under oxidative stress and then assess if the stressful environment was sufficient to significantly alter the strength of the correlation [9].

In *A. vaga* we find that under standard laboratory conditions there is a significant positive correlation between reproduction and lifespan. However, when faced with an environment known to induce oxidative stress and reduce reproduction, the correlation that describes the irradiated cohort is significantly reduced relative to the non-irradiated cohort. These results suggest that although oxidative stress does not result in a trade-off between survival and reproduction, oxidative stress does influence the strength of the correlation between these traits, and plays some role in shaping this relationship.

The baseline patterns of age-specific survival and reproduction in *A. vaga* suggests heavy investment in reproduction between the ages of 5 and 10 days. This reproductive investment is coupled with an increased mortality rate beginning around age 8 days shortly before reproduction also begins to drop-off. Combined, these results are suggestive of a cost of reproduction. Concomitant with the changes in age-specific reproduction and survival are significant increases in variables that describe the internal biochemistry related to oxidative stress. There is a significant increase in oxidative damage, as measured by levels of protein carbonylation, between 5- and 10-day-old individuals, and this elevated level of carbonylation remains through age 25 days (Fig. 1b). This observation provides correlative evidence that a cost of reproduction is elevated levels of oxidative damage, and that this sustained increase in oxidative damage after age 10 days may drive the monotonic decline in survival that begins at age 8 days.

Interestingly, the increases in oxidative protein damage are preceded by increases in levels of H_2_O_2_ (Fig. 1c). At age 5 days there is a noticeable increase in H_2_O_2_, while at age 10 days levels of protein carbonylation rapidly increase. The observation that the age-specific patterns of oxidative stress (H_2_O_2_) and oxidative damage (carbonylation) are similar in shape, but out of phase by 5 days (and perhaps less time had we included more sampling intervals), may be explained by the fact that H_2_O_2_ does not directly cause the oxidative modification of proteins of the type we measured here. Rather, H_2_O_2_ penetrates the active sites of enzymes containing iron-sulfur clusters and generates unstable iron-sulfur forms because it induces the release of ferric iron [15]. The released ferric iron, when reduced to ferrous iron, and in concert with other cytosolic ferrous iron, can then interact with H_2_O_2_ through a series of biochemical steps known as the Fenton reaction [16], which results in the formation of highly reactive hydroxyl radicals that directly attack proteins resulting in their oxidative modification. To combat the direct and indirect damage induced by H_2_O_2_ organisms often increase levels of peroxidase and catalase, which scavenge H_2_O_2_. Our results show that the age-specific patterns of peroxidase/catalase activity roughly approximate the age-specific patterns of H_2_O_2_ levels (Fig. 1d). Interestingly, the change in H_2_O_2_ from age 2 to age 25 was disproportionately larger than the change in peroxidase/catalase activity (300% increase in H_2_O_2_ levels vs. a 35% increase in peroxidase/catalase activity). Thus, the elevated levels of protein carbonylation in reproductive and post-reproductive *A. vaga* that follow peaks in H_2_O_2_ and enzyme activity may reflect increased hydroxyl radical formation via the Fenton reaction that arises due to an imbalance between endogenous levels of H_2_O_2_ and its enzymatic scavengers.

While our baseline measurements of reproduction, survival, and biochemistry suggest survival, reproduction, and oxidative stress are related, our interpretation is based on qualitative inference. To further examine the nature of this relationship we experimentally modified reproductive schedules using exposure to IR. The primary free radical generated by exposure to IR is the hydroxyl radical, which is generated through the radiolysis of intracellular water molecules. Our results suggest that when faced with the oxidative threat of excess hydroxyl radicals, *A. vaga* may prioritize somatic maintenance at the expense of germline maintenance. Specifically, age-specific patterns of survival do not differ between irradiated and non-irradiated cohorts (Fig. 2a), but the age-specific patterns of reproduction are significantly altered (Fig. 2b).

Our rationale for performing the experiments described above was that by exposing individuals to environments known to induce oxidative stress, we presumably force *A. vaga* to divert resources to mechanisms associated with the protection and repair of somatic cells, which consequently reduces the protection and repair of germline cells. Alternatively, *A. vaga* may preferentially protect and repair germline cells instead of somatic cells, resulting in a cost of reproduction in the form of increased mortality. Our results suggest that *A. vaga* is highly efficient at maintaining the soma under oxidative stress, but the cost of somatic maintenance is reduced germline protection, indicated by reduced reproduction. This conclusion contrasts our earlier suggestion that, under benign laboratory conditions where levels of free radicals are determined endogenously in the absence of exogenously applied oxidative stress, reduced survival represents a cost of reproduction. However, one explanation that may reconcile these observations is that under benign conditions, where the levels of endogenous free radicals are minimized, it is costs of reproduction in the form of increased oxidative damage that determine lifespan, while under conditions of excess oxidative stress reduced reproduction is a cost of somatic maintenance. This hypothesis is, in part, corroborated by another unique feature of bdelloid biology, their ability to survive and resume reproduction following desiccation at any life stage (i.e., anhydrobiosis) [17]. During desiccation, free radicals are generated in large quantities due to the disruption of electron-transport chains [18, 19]. As a consequence, bdelloids have evolved a highly efficient antioxidant system [11] that facilitates maintenance of a viable soma while desiccated. While in this desiccated state, reproductive activity ceases completely, and then returns to normal, or even increases, following rehydration [20]. Overall, anhydrobiotic responses in bdelloids are congruent with the notion that under conditions of elevated oxidative stress such as those that occur while desiccated, bdelloids place a premium on the protection and repair of somatic cells.

The primary defense against oxidative damage of the type applied here involves non-enzymatic small-molecule antioxidants whose primary purpose is to scavenge hydroxyl radicals. Previous studies on the role of small-molecule antioxidants in rotifer life-histories have shown that bdelloids whose food or culture medium is supplemented with vitamin E enjoy enhanced longevity [21–23] presumably due to reduced loads of oxidative damage conferred by the hydroxyl radical scavenging by vitamin E. Additionally, a screen of the effects of 20 different small-molecule antioxidants on survival in the monogonont rotifer, *Brachionus manjavacas*, showed rotifers provided antioxidants singly or in various combinations enjoyed enhanced survival when challenged with the oxidizing agent juglone, and that some combinations of antioxidants could also extend lifespan [24]. Although the composition of the small-molecule antioxidant system in bdelloids is not well-characterized [11], the results presented here suggest it is an integral component of the oxidative stress response system capable of protecting native *A. vaga* proteins from IR-induced protein carbonylation, and extending the survivable range of IR doses in bacterial cells by 33% (Fig. 3). Therefore, the biosynthesis, or acquisition, sequestration, and bioaccumulation from food, of small-molecule antioxidants may represent a source of resource investment aimed at somatic maintenance during periods of oxidative stress.

## Conclusions

Oxidative stress as a mediator of life-history trade-offs provides an attractive hypothesis to describe the physiological mechanisms that drive life-history evolution. However, even in the absence of a trade-off sensu stricto between lifespan and reproduction, significant changes in the strength of the correlation between these traits may also indicate that oxidative stress mediates life-history. In *A. vaga*, qualitative inference suggests that costs of reproduction in the form of increased mortality rates may be linked to measures of oxidative stress under benign conditions. In contrast, experimental exposure to IR, which is known to induce oxidative stress, causes a reduction in reproductive effort, but no change in patterns of survival, indicating a cost of somatic maintenance. The observation that bdelloid survival is insensitive to the harmful effects of oxidative stress may stem from their ability to undergo anhydrobiosis. Additionally, the fact that bdelloids are eutelic, both somatic and germline cells are quiescent upon hatching, and therefore lack mechanisms to replace dead cells as juveniles and adults places a premium on maintaining proper function of the existing cells under stress. Thus, resource investment into somatic maintenance is critical to maintaining proper organismal function, even if this strategy necessitates a reduction in other aspects of fitness. Finally, the core of the antioxidant system involves highly efficient small-molecule scavengers whose biosynthesis represents a sink into which resources may be diverted, and that provide protection of the soma at the expense of reproduction.

## Methods

### Baseline patterns of age-specific changes in life-history and biochemistry

#### Survival and reproduction

As a model system for this investigation we employed a clonal lineage of *A. vaga* that has been extensively utilized in previous studies that have examined, for example, the genome architecture of bdelloid rotifers [25] and bdelloid sensitivity to IR [10, 11, 26]. A life-table experiment was conducted to establish the baseline age-specific schedules of survival and fecundity under the benign laboratory conditions. Seventy-two individuals were isolated from stock cultures and maintained individually in 120 µL drops of sterile spring water (Poland Spring^®^ bottled water autoclaved for 1 h at 121 °C) mixed with *E. coli* as a food source on lids of a 96-well microplate at 20 °C in a 12:12 light:dark photoperiod. We minimized evaporation from the drops by placing the lids inside a sealed plastic container with a reservoir of sterile spring water to maintain elevated humidity. Additionally, to minimize changes in drop volume, and to maintain constant food levels we replaced the spring water/food mixture every other day. The *E. coli* used as the food source was propagated by initiating liquid cultures from stock plates maintained at 4 °C in 1 L of liquid LB medium maintained at 37 °C overnight and subsequently concentrated by centrifugation. The concentration of food in the spring water/food mixture was 2 × 10^7^ cells of live *E. coli* strain M28 per 120 µL water.

To minimize variance due to maternal and grand-maternal environmental effects single individuals were maintained under these constant conditions for two generations prior to the life-table experiment. Third-generation individual rotifers were placed in a life-table experiment and monitored daily for survival and fecundity. Individuals were considered dead when they were no longer responsive to gentle agitation and touch with a micropipette. Fecundity was estimated by counting the number of eggs deposited. After counting, these eggs were removed by micropipette. To ensure minimal volume changes in the drops, eggs were removed prior to culture medium replacement, or, on alternate days when culture medium replacement was not conducted, eggs were removed in volumes of 1 µL/egg. Since no more than 5 eggs were deposited on any day, the total change in water volume due to egg removal was no more than 4% of the total volume.

#### Generation of even-age cultures

Our first goal was to acquire age-specific estimates of the oxidative stress biochemistry that we could correlate with the patterns of survival and fecundity we measured in the life-table experiment. To accomplish this goal we generated even-age cultures by placing stock cultures on ice for 1 h and then transferred the culture to a new 150 mm × 25 mm Petri dish. Placing cultures on ice prior to transfer induces juveniles and adults to form tuns (compact body forms induced by exogenous stress [27]), and prevents them from adhering to the surface of the new plate, while eggs from the stock culture adhere to the new plate. The transferred culture was allowed to sit for 15 min to allow egg adherence and then the culture was transferred back to the stock plate leaving behind a new plate with predominantly eggs. To remove residual juveniles and adults that did adhere to the new plate, plates were washed with 10% ethanol, which also induces tun formation, and then rinsed twice with cold spring water. This procedure was repeated twice.

After juveniles and adults were removed with ethanol and spring water rinses, 200 mL of spring water supplemented with 1 mL of concentrated live *E. coli* was added to the new plate. One-day old hatchlings were isolated from the new plate 24 h later by placing on ice for 1 h and then transferring the supernatant containing hatchlings to a new dish. When individuals matured and began to deposit eggs, the adults were separated from the eggs by placing the plate on ice for 1 h, to induce tun formation in adults, and transferring the supernatant to a new plate every day until a culture containing individuals of homogenous age was achieved. In order to perform biochemical assays on different age classes at the same time we initiated even-age cultures using the procedure described above every 5 days, or 3 days in the case of the youngest age class, so that even-age cultures corresponding to ages 2, 5, 10, 15, 20, and 25 days old could be harvested for biochemical extraction simultaneously. From this set of cultures we obtained enough tissue to perform five replicates from each age class for the protein carbonylation assay, and four replicates for the H_2_O_2_ and catalase/peroxidase assays.

#### Generation of extracts for biochemical analysis

Extracts for biochemical assays were generated by placing cultures on ice for 1 h to induce tun formation, and then the supernatant was filtered through 5 µm nitex mesh under a light vacuum with periodic cold spring water rinses. Individuals were then transferred from the nitex to a 50 mL conical tube and centrifuged at 3500 rpm for 5 min. After centrifugation, the supernatant was removed and 25 mL of cold 1X PBS were added followed by centrifugation at 3500 rpm for 5 min to further rinse the pellet. The pellet was then transferred to a 1.7 mL microcentrifuge tube, centrifuged at 3500 rpm for 5 min, and the supernatant removed. Extraction buffer consisting of cold 1X PBS supplemented with a protease inhibitor cocktail (Pierce Biotechnology, Rockford, I L) was then added to the pellet. The tubes were subjected to two liquid nitrogen freeze–thaw cycles and Dounce homogenization. Tubes were then centrifuged at 14,000 rpm for 12 min and the supernatant removed and transferred to a new 1.7 mL microcentrifuge tube. Because the number of individuals collected to generate extracts varied according to the age class (from ~ 1000 individuals for age 25 extracts to ~ 10,000 individuals for the age 2 extracts), we standardized each extract for protein content by first quantifying protein content with the BCA assay (Bio-Rad, Hercules, CA), performed according to manufacturer instructions, and then dilution with 1X PBS with protease inhibitor.

#### Protein carbonylation

In order to obtain age-specific measures of the amount of oxidative damage in *A. vaga* maintained in a benign environment we measured protein carbonylation. Carbonylation data were collected from two independently generated sets of even-age cultures using the OxiSelect Protein Carbonyl ELISA Kit (CellBiolabs Inc., San Diego, CA) according to the manufacturer’s protocol.

#### Hydrogen peroxide

As a measure of the amount of endogenous oxidative stress *A. vaga* experiences with age we quantified levels of H_2_O_2_. Levels of H_2_O_2_ in cytosolic extracts derived from the same independently generated sets of even-age cultures were assayed using a protocol derived from the Invitrogen Amplex Red Hydrogen Peroxide/Peroxidase assay kit (Cat. No. A22188), substituting Ampliflu Red (Sigma). Hydrogen peroxide standard curves were generated using a laboratory stock solution serially diluted in 1X (50 mM) NaH_2_PO_4_. Fifty to 100 µL of samples and standards were added to the wells of a 96-well microplate and 50 µL of working solution (100 µM Ampliflu Red, 0.2 U/mL horseradish peroxidase in 50 mM NaH_2_PO_4_) were added to each well. Following a 30 min incubation at room temperature, fluorescence emission was assayed at 590 nm with excitation at 530 nm and optimal gain on a Tecan Infinite M200 Pro plate reader.

#### Catalase/peroxidase activity

We assessed the age-specific levels of oxidative stress resistance due to enzymatic processes in *A. vaga* by measuring the combined ability of catalase and peroxidase to remove H_2_O_2_ from solution. The ability of extracts to remove H_2_O_2_ from solution was assayed using an amplex red catalase assay protocol derived from that used with the Invitrogen kit (A22180) but substituting Ampliflu Red (Sigma) and laboratory stock reagents (i.e., anhydrous dimethylsulfoxide, horseradish peroxidase, 3% H_2_O_2_, 0.5 M Tris–HCl at pH 7.5, and catalase). Standard curves for catalase activity were generated using purified enzyme (Sigma) serially diluted in 1X reaction buffer (Tris–HCl). Twenty-five microliter of 40 µM H_2_O_2_ was added to 25 µL of each standard or sample in a 96-well plate and incubated for 30 min at room temperature, after which 50 µL of amplex red working solution was added and incubated for an additional 30 min at room temperature. Fluorescence emission was assayed at 590 nm with excitation at 530 nm and optimal gain on a Tecan Infinite M200 Pro plate reader.

### Life-history and biochemistry following acute exposure to ionizing radiation

#### Survival and reproduction

One method of manipulating fecundity, and possibly the relationship between survival and reproduction, in *A. vaga* is to expose individuals to IR [11]. We isolated pre-reproductive individuals (1–4 days old) from stock cultures of mixed age and generated two groups for common garden experiments. One group of 46 individuals was placed on ice and exposed to a dose of 1200 Gray (Gy) of IR, a dose previously shown to reduce fecundity but not survival [11], using a ^137^Cs source at a dose rate of 270 Gy/h. The second group of 18 individuals was placed on ice but not exposed to IR. Following irradiation, individuals from each treatment were used as cohorts in life-table experiments using the same protocol as outlined previously except that individuals were not passed through two generations prior to measurement. The rationale for using a small sample of non-irradiated individuals in parallel with the irradiated cohort, despite previously establishing patterns of survival and reproduction for non-irradiated individuals during our baseline assays, was twofold. First, the cohort used in baseline life-table experiment was not conducted simultaneously with the cohort used for the IR life-table experiment, so we wished to start a small non-irradiated cohort to run simultaneously with the IR cohort. Second, both cohorts (irradiated and non-irradiated) used in the IR experiments were not passed through two generations prior to measurement, so using a small non-irradiated cohort for comparison with the irradiated cohort minimizes the potential confounding influence of transgenerational effects that may have been present had we directly compared the cohort from the baseline assays to the irradiated cohort.

#### Oxidative stress biochemistry in response to ionizing radiation

Exposure to IR generates hydroxyl radicals that directly cause protein and DNA oxidation and can only be scavenged by small-molecule antioxidants. In order to examine the ability of this non-enzymatic system of oxidative stress resistance to protect *A. vaga* from hydroxyl radicals we examined levels of protein carbonylation in extracts differing in composition. We generated protein extracts using the protocol outlined above. After extraction, half of each extract was subjected to centrifugal filtration through a 3 kD MW spin column and the filtrate collected. We also resuspended the protein collected in the 3 kD spin column filter using a volume of PBS/protease inhibitor equal to the amount originally filtered. Thus, we had three types of extracts, extract containing both small molecules and proteins (whole extract), extracts with only small molecules (filtrate), and extracts with only proteins (high molecular weight protein extract).

We measured the ability of *A. vaga* extracts to protect their native proteins from protein carbonylation induced by IR using the OxiSelect Protein Carbonyl ELISA Kit. Whole extracts and high molecular weight protein extracts were irradiated at doses corresponding to 0, 500, 1000, and 2000 Gy. The high molecular weight protein extracts provide a means to assess the total carbonylation of native proteins in *A. vaga* without small molecule protection, while the whole extracts indicate the level of carbonylation with small molecule protection.

As an additional method to test the ability of the non-enzymatic oxidative stress resistance of *A. vaga* to protect against hydroxyl radicals we used whole extracts and filtrates to measure the ability of extracts to protect live cells. The *E. coli* strain OP50 was cultured in liquid LB medium overnight at 37 °C. The culture was then pelleted with centrifugation, washed with ddH_2_0, and serially diluted 125,000X in 1X PBS containing 0.06 M MgSO_4_. Fifty microliter of the diluted cell suspension was added to 250 µL of 1X PBS/protease inhibitor, *A. vaga* whole extract, or 3 kD filtrates standardized for protein content. The solutions containing *E. coli* cells were then irradiated and aliquots were removed from each sample at doses corresponding to 250 Gy, 375 Gy, and 500 Gy. Cell survival was estimated using a colony forming unit (CFU) assay where three replicates of 300 µL from each extract were plated on LB plates, incubated overnight at 37 °C, and colonies counted following incubation.

### Statistical analysis

All data used for the analyses described below are contained in Additional file 1.

#### Baseline

Survival data from the common garden experiment were fit to a Gompertz mortality model to obtain an estimate of the mortality rate doubling time using the Survomatic package [28] in Program R [29]. Survival and fecundity data were considered jointly to estimate the intrinsic rate of increase. Biochemical assays were subjected to one-factor ANOVA with a fixed main effect of age (6 levels) followed by post hoc pairwise comparisons using the Tukey method of p-value adjustment to account for multiple comparisons in Program R.

#### Ionizing radiation

To compare survival curves of the treated and untreated cohorts we used Cox proportional hazards regression [30]. Age-specific fecundity for the treated and untreated cohorts was compared using a generalized linear mixed effects model under maximum likelihood assuming a Poisson distribution for the offspring counts using the lme4 package [31] in Program R. In the model, treatment, age, and age^2^ (the squared term is included to account for the non-linearity in age-specific fecundity across age classes) were modeled as fixed effects while individuals were modeled as a random effect. Protein carbonylation was analyzed with two-factor ANOVA with extract (either whole or high molecular weight protein) and IR dose treated as main effects. The *E. coli* CFU assay was analyzed with a generalized linear model assuming a negative binomial distribution for the colony counts by treating extract (PBS only, whole, or filtrate) and IR dose as fixed main effects and plate as a random effect.

## Additional file

**Additional file 1.** The raw data used for the analyses described in the manuscript.

## Abbreviations

IR: ionizing radiation
PBS: phosphate buffered saline
ELISA: enzyme-linked immunosorbent assay
BCA: bicinchoninic acid
ANOVA: analysis of variance
Gy: grays
CFU: colony-forming unit
